# African leaders and regional institutions need to take the bull by the horns: a perspective on the impact of the 2025 funding cuts to malaria programmes

**DOI:** 10.1186/s12936-025-05736-5

**Published:** 2025-12-18

**Authors:** Caroline B. Osoro, Jenny Hill

**Affiliations:** 1 Population and Health Impact Surveillance Group, KEMRI-Wellcome Trust Research Programme, Nairobi, Kenya; 2 Department of Clinical Sciences, Liverpool School of Tropical Medicine, Liverpool, UK

**Keywords:** Global health, Malaria, Africa, Health financing

## Abstract

Despite global malaria programmes already operating within resource constraints, 2025 saw a significant decrease in funding following the US government’s termination of most of its global health programmes, as well as the decline in development aid spending by the UK, France, Germany, Canada, Switzerland, and other countries. The disruption of funding was sudden, with most African countries lacking adequate contingency plans. This, despite most of the malaria burden being in Africa (94% of 263 million cases in 2023), accounting for a reduction in gross domestic product of up to 1.3% annually, and half a billion lost workdays. Key malaria control programme activities have been severely impacted, including insecticide-treated bed net distribution, seasonal malaria chemoprevention campaigns, and malaria indicator surveys. In the wake of the funding cuts, some African governments have committed to increasing efforts to raise funds for malaria programmes from the private sector. The Africa Centres for Disease Control and Prevention (CDC) has developed a strategy for governments to increase health budgets while seeking additional funding from the private sector, all while maintaining transparency and accountability. If recent malaria control gains are to be sustained and to prevent resurgence across the continent, African governments will need to increase domestic funding and build robust public–private partnerships for their malaria programmes. Lessons can be learnt from countries where these partnerships have succeeded or failed. Leadership by the African Union, the Africa CDC, the African Leaders Malaria Alliance, and other regional bodies is crucial to support countries in taking immediate, substantive steps and benchmarking progress.

## Background

The year 2025 brought an abrupt disruption to international funding for global malaria programmes, which were already operating under significant resource constraints [1]. In February, the US government announced foreign aid cuts, starting with the immediate termination of 86% of programmes at its Agency for International Development (USAID) [2]. It subsequently announced the sudden termination of funding to GAVI, the Vaccine Alliance—a public–private partnership that helps vaccinate more than half the world’s children—and a full review of its support to international organizations, including the Global Fund for AIDS, TB and Malaria, the World Health Organization (WHO), the Green Fund and the World Bank, all key funders of malaria control programmes [3] [4]. The US government, through its proposed fiscal year 2026 budget, plans a further 62% cut to funding for global health programmes, a 45% cut to the President’s Malaria Initiative, and zero allocations to the Global Fund for AIDS, TB and Malaria and GAVI [5]. The impact of these measures, taken without warning, has been devastating. Put into context, in the financial year 2024, the US government provided $12.3 billion in global health appropriations; funding 33% of the Global Fund budget, 20% of the WHO budget, 15% of the GAVI budget, 22% of the Green Fund and 16% of the World Bank budget [6] [7]. The US government is not alone. Other government agencies have announced funding cuts for global health programmes. The UK announced in March 2025 that its aid budget would be reduced from 0.5 to 0.3% of gross national income from 2027 [8]. France, Germany, Canada, Switzerland, and other countries have also announced reductions in aid spending [9]. Consequently, the Global Fund, which provides 59% of all international financing for malaria programmes, issued guidance on the reprogramming of its activities due to a shortfall in its 2023–2025 grant cycle [10]. In its eighth replenishment for the 2027–2029 cycle, the Global Fund raised only $11.34 billion of its planned $18 billion [11]. Subsequently, these government cuts have put greater pressure on philanthropic organizations, such as the Gates Foundation, which has provided over $100 billion in the last 25 years, announcing in May 2025 that it would permanently close its doors in December 2045 [12]. The news came swiftly and unexpectedly, with most African countries lacking contingency plans.

## Malaria: a looming public health and economic crisis

While significant gains have been made since 2000, malaria continues to be a major public health concern, with 263 million cases globally in 2023, 94% of which were in the WHO Africa region [13]. Yet, these numbers tell only part of the story. The disease impacts national productivity and strains the already resource-constrained health systems in many African nations. In malaria-endemic countries in Africa, the disease is reported to have resulted in a reduction of gross domestic product (GDP) of up to 1.3% annually, with half a billion lost workdays [14] [15]. With a dramatic decline in procurement, Africa risks losing its manufacturing capabilities for pharmaceuticals and malaria commodities, leading to job losses and further impacting national economies. While malaria programmes are already facing challenges with drug resistance and demand exceeding supply for malaria vaccine roll-out, cessation or even a reduction in control activities would lead to a resurgence of malaria across the continent causing catastrophic economic decline. This, along with the impending threat of conflict and climate change, is predicted to reverse some of the gains already made in malaria control, creating the perfect storm [16] [17].

## Implications of funding cuts

The 2025 funding cuts have resulted in disruption of: (1) health services leading to facility closures, (2) malaria commodity supply chains, (3) the roll-out of the new malaria vaccine, (4) indoor-residual spraying and insecticide-treated net (ITN) distribution campaigns, and (5) surveillance activities, such as demographic health surveys, national malaria indicator surveys, drug and insecticide resistance monitoring [6] [18]. More than half of Africa’s 64 malaria-endemic countries have reported disruptions to malaria services; 40% of planned ITN distribution campaigns targeting 425 million people have been delayed, and 30% of seasonal malaria chemoprevention campaigns to protect 58 million children have been compromised [19]. Furthermore, most African countries report critically low levels of malaria commodities, including rapid diagnostic tests and antimalarials [19]. This is all happening against the backdrop of an extraordinary demand for the malaria vaccine, which has been introduced in 24 African countries as of November 2025 [20]. Most of these countries have only recently introduced the vaccine, while a few are looking to scale up from pilots, both processes requiring extensive technical and financial support [16] [21]. Malaria indicator surveys, which were planned for 2025 in Uganda, Kenya, and Nigeria, have stalled, which will affect evidence-based decision-making for targeting increasingly limited malaria programmes and commodities [18]. Critically, the cuts have also targeted and deprioritized research and innovation, which are crucial to the continued advancement of malaria control and progression towards elimination. Even in countries where malaria elimination has been achieved, governments need to maintain surveillance, case detection and treatment to prevent reintroduction. A rebound in malaria, given countries’ lack of capacity to implement preventive interventions, is estimated to cost Africa $402 billion by the year 2040 [10].

## Action taken by governments and regional institutions

**Fig. 1:**
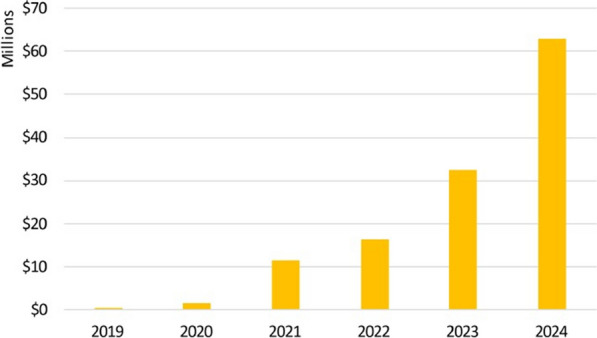
Resources mobilized by End Malaria Councils and Funds by year (Source: The African Leaders Malaria Alliance (ALMA) 2024 Malaria Progress Report)

In the immediate aftermath of the announcement of the US government aid cuts, one country, Nigeria, approved an additional $200 million for its health sector, against its $600 million deficit [22]. Countries also sought to increase public–private financing partnerships for malaria programmes. Successful countries in this endeavour include Uganda, Eswatini, Kenya, Mozambique, Nigeria, Tanzania, and Zambia, which, through the African Leaders Malaria Alliance (ALMA), committed to increasing efforts to raise funds from the private sector, having so far mobilized $125 million in cash and kind [23] [24]. However, the call for African governments to tap into private sector funding and enhance public–private partnerships predates the 2025 funding fiasco. Before the periodic replenishment of funds for organizations such as the Global Fund, concern has been raised about the sustainability of malaria control programmes should these organizations fail to meet their targets. It is critical that regional institutions, including the ALMA, End Malaria Councils and Funds (EMCs), and the African Union (AU), through its public health agency, the Africa Centres for Disease Control and Prevention (CDC), step up and guide country efforts during these challenging times. EMCs are multi-sectoral, country-owned, and led forums. Notably, the EMCs of four countries (Mozambique, Tanzania, Uganda, and Zambia) have made efforts to pool and distribute resources from new donors and the private sector, raising $62 million in financial and in-kind support in 2024, as well as pro-bono technical expertise (Fig. 1). Other African countries have expressed interest in establishing EMCs, with Burkina Faso and Liberia launching in April 2025.

The Africa CDC’s April 2025 health financing strategy to mitigate the impact of decreased foreign aid is another worthy step. The strategy’s three pillars are increased domestic funding, more innovative funding, and blended financing that combines public and private sector capital [25]. Further, the strategy aims for African countries to increase health budgets, including an update of health financing plans in 30 countries, scale innovative financing with a target of achieving 50% domestic financing for health in 20 countries and develop transparency scorecards and dashboards [25]. As African governments look to enhance public–private financing partnerships, lessons must be learnt from countries where this has been a success. Countries with operational EMCs, including Kenya, Mozambique, Zambia, Ghana, and Eswatini, have demonstrated that financing partnerships flourish when there is strong government ownership, transparent financial management, and robust reporting and monitoring structures [26]. Conversely, financing partnerships have not been as successful in countries such as Burkina Faso, where the government has opposed external funding for some malaria control activities [27]. Transparency in financial management is a significant hurdle in most countries, and the transparency scorecards and dashboards proposed in the Africa CDC’s health financing strategy may improve accountability.

As external funding sources shrink, African governments must plan for resilient health systems that are funded primarily by domestic budgets and robust public–private partnerships. Malaria is just one of the public health challenges in Africa, with the other major contributors to morbidity and mortality (HIV/AIDS, Tuberculosis and non-communicable diseases like cardiovascular diseases, diabetes and cancer) also competing for the scarce resources [28]. A rethinking of malaria control programmes is needed. First, enhanced integration of malaria programmes within the broader health system is called for, including integrated financing within primary health care. Primary health care has been identified as an essential organising framework for health systems strengthening in Africa [29]. Some examples of integration include: countries’ inclusion of the malaria vaccine into routine immunisation schedules [30] and Kenya’s planned integration of malaria case management into primary health care services and alignment with maternal and child health services [31]. However, the integration of malaria programmes must be done judiciously to avoid compromising the core mandate of primary health care, which is to provide basic care and interventions [32]. Second, governments in higher-income African countries must find ways to absorb the costs of health infrastructure and human resources that external funding sources have previously supported, recognizing this may be more challenging in the lowest-income countries. To achieve this, governments can create new or leverage existing EMCs to create a platform of multi-sectoral financiers. Third, malaria commodity supply chains must be maintained to prevent resurgence. This could be through integration with national logistics and procurement systems and support of locally manufactured malaria commodities [15]. Fourth, surveillance activities need to be prioritized as data is critical for planning and cost-effective resource allocation. Following the announcement of the US aid cuts, some countries changed their surveillance systems to focus on sentinel sites and hot-spots and collected primarily programmatic indicators. However, moving forward, malaria programmes can make better use of the existing District Health Information Software 2 (DHIS2). Current data on malaria indicators and robust commodity management are essential for allocating scarce resources effectively. Surveillance data on the effectiveness of existing interventions is critical, as it will inform continued research and innovation for new alternatives. National malaria control programmes will need to establish and drive the utilisation of limited resources in line with national priorities, using stratified approaches that maximize the cost-effectiveness of tool combinations.

## Conclusion

With the African Union aiming to achieve a 100% reduction in malaria incidence and mortality by 2030, the continent faces a monumental challenge. The African Leaders Malaria Alliance, in its 2024 progress report, states that ‘*defeating malaria by 2030 could yield a 40 to 1 return on investment, making it one of the highest-impact investments in global health*’ [15]. To this end, the continent must seriously consider increasing its spending on research and development activities from the current 0.5% of GDP, compared to the world average of 2.2% [33]. It is time for African governments to make decisive investments in their malaria control programmes, and leadership by the AU will see countries take immediate and substantive steps.

## Data Availability

No datasets were generated or analysed during the current study.

## Abbreviations

Africa CDC: Africa Centres for Disease Control and Prevention
ALMA: African Leaders Malaria Alliance
AU: African Union
EMC: End Malaria Councils and Funds
GAVI: The Vaccine Alliance
GDP: Gross domestic product
ITN: Insecticide-treated net
UK: United Kingdom
USA: United States of America
USAID: United States Agency for International Development
WHO: World Health Organization
